# Group Vaccination Five Days before a COVID-19 Outbreak in a Long-Term Care Facility

**DOI:** 10.3390/vaccines9121450

**Published:** 2021-12-08

**Authors:** Mathias W. Pletz, Sabine Trommer, Steffi Kolanos, Norman Rose, Veit Kinne, Riccardo Spott, Michael Baier, Isabel Lange

**Affiliations:** 1 Institute of Infectious Diseases and Infection Control, Jena University Hospital, Friedrich Schiller University, 07747 Jena, Germany; Steffi.Kolanos@med.uni-jena.de (S.K.); norman.rose@med.uni-jena.de (N.R.); Veit.Kinne@med.uni-jena.de (V.K.); Riccardo.Spott@med.uni-jena.de (R.S.); Isabel.Lange@med.uni-jena.de (I.L.); 2 Center for Sepsis Control and Care, Jena University Hospital, Friedrich Schiller University, 07747 Jena, Germany; 3 Public Health Department, City of Jena, 07743 Jena, Germany; Sabine.Trommer@jena.de; 4 Institute of Medical Microbiology, Jena University Hospital, Friedrich Schiller University, 07747 Jena, Germany; michael.baier@med.uni-jena.de

**Keywords:** COVID-19, outbreaks, mass vaccination, long-term care facility, pre-symptomatic patients

## Abstract

Rapid vaccination may be of benefit in long-term care facilities (LTCF) that are affected by an ongoing COVID-19 outbreak. However, there are concerns regarding the safety and effectiveness of such an approach, particularly regarding the vaccination of pre-symptomatic patients. Here, we report the effectiveness of vaccination in a German LTCF hit by an outbreak that was detected 5 days after the first vaccine doses were administered. In detail, 66.7% of the unvaccinated patients experienced an unfavorable course; this proportion was much lower (33.3%) among the vaccinated patients. Even though this study is limited by a small number of patients, the observation and the comparison with related published data shows that vaccination (i) is safe and (ii) may still be of benefit when given shortly before an infection or even in pre-symptomatic LTCF-patients.

## 1. Introduction

COVID-19 (coronavirus disease 2019) outbreaks in long-term care facilities (LTCF) can last for weeks, are difficult to contain, and are associated with high mortality. A recently published UK-wide cohort study involving 907 cases of SARS-CoV-2 (severe acute respiratory syndrome coronavirus type 2) infection from 69 LTCF outbreaks reported a mortality of 48% [1]. A current US-study regarding the epidemiology of COVID-19 in a local LTCF confirmed, for 101 residents affected, a hospitalization rate of 54.5% and a mortality rate of 33.7% [2]. COVID-19 infections have also been detected in healthcare workers and visitors in this context [2]. ECDC (European Center for Disease Prevention and Control) reported that 30–60% of all COVID-19 deaths in Europe were patients of LTCFs [3]. They have a higher risk of hospitalization and mortality due to age and co-morbidities such as heart disease, diabetes, COPD and others [4].

The reasons why LTCFs are particularly vulnerable to SARS-CoV-2 outbreaks are poorly understood. Most of the previous studies of risk factors for SARS-CoV-2 infections in LTCFs have been limited by scope and poor quality of administrative, demographic, and infection control data [5]. However, a lower staff per patient ratio, necessities of many contact-nursing procedures, and patients who are not be able to follow hygiene instructions explain difficulties in containing LTCF outbreaks compared to hospital outbreaks. Cross-sectional study findings highlight the key role of staff, adherence to disease control measures and new admissions to LTCF in the transmission of SARS-CoV-2 infections [5]. Strategies that might reduce staff transmission of SARS-CoV-2 infections may be considered in this context. Such strategies might include adequate sick pay, minimising the use of temporary staff, improving the staff-to-bed ratio, and cohorting staff with either infected or uninfected residents [5].

The average incubation time of 5–7 days and the observation that such outbreaks can go on for weeks points toward a potential role for rapid vaccine prophylaxis in LTCF outbreaks. A Scottish prospective cohort study, analyzing a total of 1,331,993 people with a mean age of 65.0 years (SD 16.2) vaccinated between 8 December 2020, and 22 February 2021, found that the first dose of the BNT162b2 mRNA vaccine was associated with a vaccine effect of 91% (95% CI 85–94) for reduced COVID-19 hospital admission at 28–34 days post-vaccination [6]. An Israelian comparative effectiveness study of 503,875 individuals who received one dose of the BNT162b2 vaccine, the first dose of the vaccine was associated with an approximately 51% reduction in the risk of SARS-CoV-2 infections at 13 to 24 days after immunization compared with 1 to 12 days after vaccination [7]. The Kaplan–Meier curve of this publication shows a substantial reduction of new onset infections about one week after vaccination compared to unvaccinated persons.

The State of Thuringia has implemented mobile vaccination teams for LTCF. In addition to regular appointments with mobile vaccination teams, LTCF can report the first case of COVID-19 to a telephone hotline and immediately order a mobile vaccination team for vaccination of non-symptomatic patients in the respective LTCFs.

However, the minimal duration required for relevant effectiveness after the first administration of a vaccine against SARS-CoV-2 is a matter of debate and further investigation—particularly in the immunosenescent frail and elderly—and questions such an approach [8,9]. 

## 2. Materials and Methods

### 2.1. Study Cohort, Outbreak and Timeline

Here, we report the effectiveness of vaccination in a German LTCF with 29 patients (median age 85, median Frailty Score 7, range 6–8) in the city of Jena [10]. This LCTF was hit by an outbreak that was detected 5 days after the first vaccine doses were administered. Infection control interventions were masks worn by the staff, testing via antigen tests when patients show COVID-19-like symptoms, a general visitor prohibition and mandatory antigen testing when entering the facility. Given the incubation time for COVID-19 and the usual delay until outbreaks are detected, we assume that some patients were already infected at the time they received the first vaccine dose.

In this LTCF, the first vaccinations with the BNT162b2 mRNA vaccine were given to 22 of 29 patients on 8 January 2021. No other vaccine was used for the patients. Seven patients were not vaccinated because consent was not available from the next of kin. Five days later, the first symptomatic COVID-19 case was reported on 13 January 2021 via antigen testing in a vaccinated patient. In this patient, infection was potentially acquired during a prior hospital stay until the 5th of January. 

On the 19th of January, a team of the Public Health Department of the City of Jena visited the LTCF and screened all patients via nasopharyngeal swabs and PCR confirming infection in 20 of the 29 patients. Four additional patients tested positive during the second screening on 25 January.

### 2.2. Sequencing

For two isolates, Oxford Nanopore Technologies (ONT)-sequencing was performed using the ZymoResearch Quick-RNA Viral (spin-column) Kit. The library was sequenced using a MinION Sequencer for 72 h or until no sequencing activity was observed, using a R9.4.1 flow cell (FLO-MIN106). The sequencing run was controlled via the MinKNOW software with active channel selection enabled and basecalling deactivated. Analysis of the resulting data was performed using the program “poreCov” (Version 0.4.0) [11].

### 2.3. Statistics

We considered two outcomes. First, the laboratory-confirmed infection with COVID-19, and, second the unfavorable course of a COVID-19 infection, which was defined by hospitalization or death of the patients. Fisher’s exact test was used for statistical comparisons of the two groups of vaccinated and unvaccinated individuals. We also report percentages with 95% Wilson score confidence intervals and odds ratios (OR) with 95% logit confidence intervals. All statistical analyses were conducted using the free statistical software R. 

Due to the small sample especially of the control group of unvaccinated individuals, the statistical power was low. Therefore, we additionally compared the mortality of the vaccinated COVID-19 cases in our study with the mortality of unvaccinated COVID-19 cases reported by Burton et al. [1]. Differences in mortality were tested using Fisher’s exact test. We also report odds ratios (OR), the relative risk, and the risk difference with 95% confidence intervals. 

## 3. Results

**Figure 1:**
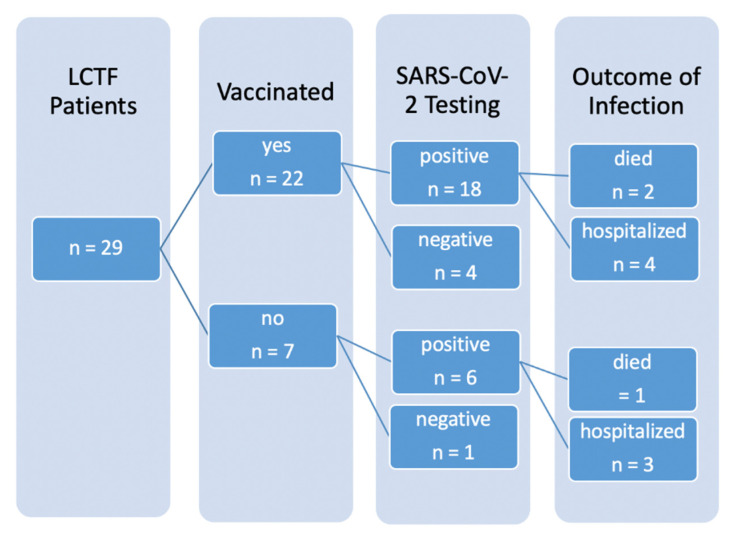
Patient flowchart.

In this outbreak, 24 of 29 patients (82.8% [65.5%, 92.4%]) tested positive (one per antigen testing, 23 per PCR), seven patients (24.1% [12.2%, 42.1%]) required admission to hospital due to COVID-19 (see Figure 1) and three patients died (10.3% [3.6%, 26.4%]). Of the seven hospitalized, three were not vaccinated. Altogether, five patients remained PCR-negative, four of them were vaccinated. The only non-vaccinated PCR-negative patient was completely bedridden and permanently isolated.

Whole genome sequencing was performed via Nanopore in two of the isolates [9]. One isolate did not meet the quality criteria, the other was identified as B.1.1.317, which is the “Russian lineage” and not considered a variant of concern [12]. Spike mutations in this isolate are S:S477N, S:A522S, S:D614G, S:Q675R.

The two groups of vaccinated and unvaccinated individuals did not differ in the proportion of confirmed COVID-19 infections (unvaccinated: 85.7% [48.7%, 97.4%], vaccinated: 81.8% [61.5%, 92.7%], OR = 0.750 [0.070, 8.089], *p* > 0.999). Infected patients in this outbreak were at high risk for an unfavorable course due to age and Frailty Score. Indeed, 66.7% [30.0%, 90.3%] of the unvaccinated patients experienced an unfavorable course. This proportion was much lower (33.3% [16.3%, 56.3%]) among the vaccinated patients. Hence, despite the short period between the first vaccination and detection of infection, two thirds of the vaccinated patients in our sample experience only mild symptoms and were treated as outpatients. However, this difference failed statistical significance (*p* = 0.192, OR = 0.259 [0.035, 1.775]) due to the small sample size.

The largest report on COVID-19 mortality in non-vaccinated patients of LTCF is the above-mentioned UK study by Burton et al. [1]. Compared with these data, the mortality of 11.1% [3.1%, 32.8%] among vaccinated individuals in our study was considerably lower than the mortality of 47.6% [44.3%, 50.8%] in unvaccinated care home residents reported by Burton et al. A statistical comparison of the mortality rates of the two studies using Fisher’s Exact Test yielded statistically significant difference (*p* = 0.002, risk difference −36.5% [−45.4%, −14.6%], OR = 0.138 [0.015, 0.592], relative risk = 0.234 [0.063, 0.864]). Hence, the mortality in vaccinated individuals with COVID-19 was 76.6% [0.136%, 93.7%] lower compared to unvaccinated COVID-19 cases in the LTCF patients described by Burton et al. [1].

## 4. Discussion

Our findings suggest that, although the first administration of a vaccine against SARS-CoV-2 shortly before or during incubation of a COVID-19 infection is not sufficient to prevent symptomatic disease, particularly in immunosenescent elderly individuals, the course of a COVID-19 disease may be positively affected by a vaccination. There was also no evidence of aggravation of an incubating infection by vaccination. As many outbreaks in LTCFs can last for several weeks, targeted vaccination in these facilities, even after the first cases are known, seems to be a safe and helpful measure to reduce the high mortality associated with these outbreaks. 

In the interpretation of the findings, limitations must be taken into account. First, the data stem from an observational study. Hence, the group of vaccinated and non-vaccinated individuals may differ systematically regarding potentially confounding covariates. This limits causal interpretation of the reported group differences. This is also true for the between-study comparisons of the mortality rates of the vaccinated individuals in our study with the unvaccinated care home residents reported by Burton et al. Potential differences in relevant covariates between the samples of both studies including possible differences in patient characteristics, health care structure and virus variants. Second, the results of our study rely on data from a single LTCF, which limits generalizability. Alternative study designs, such as randomized controlled trials, or comprehensive covariate adjustment methods (e.g., propensity score methods) in prospective studies with larger samples (i.e., more LTCFs as well as more individuals) are required to support the findings in this study. However, investigating the described constellation in a proper study is difficult. Therefore, to date, respective data are scarce. The very limited number of patients and the heterogeneity do not permit an effectiveness vaccine evaluation.

## 5. Conclusions

This outbreak report is limited by a small number of patients, which did not reveal a statistically significant difference between vaccinated and non-vaccinated patients. However, this observation and the comparison with related published data shows that vaccination (i) is safe and (ii) may still be of benefit when given shortly before an infection or even in pre-symptomatic LTCF-patients. Therefore, the findings of our study suggest that rapid vaccination in LTCF can attenuate an acute COVID-19 outbreak. 

The outbreak of COVID-19 within LTCFs is unavoidable without thorough screening of employees and visitors of LTCFs. Therefore, regular testing of those persons should help to reduce the number of outbreaks of COVID-19. Furthermore, standardized hygiene concepts implemented in LTCFs could help to prevent further spread of COVID-19. However, these measures should not be considered as complete and are subject to regular and further investigations.

## Data Availability

The data presented in this study are available on request from the corresponding author.
